# Avoiding psychological (re)traumatisation in dentistry when working with patients who are adult survivors of child sex abuse

**DOI:** 10.1038/s41415-022-5103-z

**Published:** 2022-10-28

**Authors:** Susanna Alyce, Danny Taggart, Indiana Montaque, Jackie Turton

**Affiliations:** 414157874001 PhD Candidate, School of Health and Social Care, University of Essex, Essex, UK; 414157874002 Senior Lecturer, School of Health and Social Care, University of Essex, UK; 414157874003 DClinPsy Candidate, School of Health and Social Care, University of Essex, UK; 414157874004 Emeritus Professor, Department of Sociology, University of Essex, UK

## Abstract

**Introduction**Seven percent of the adult population in the UK, including one in six women, report unwanted sexual experiences before the age of 16. The impacts of psychological trauma following child sexual abuse (CSA) creates difficulties for many survivors in accessing dental care due to fears of reminders of abuse, the power imbalance with the dentist and triggered traumatic responses.

**Aims**To analyse and report CSA survivor perspectives of dental care and offer suggestions for practice.

**Method**Qualitative semi-structured interviews of 17 CSA survivors generated data as part of a broader study investigating trust and trustworthiness in survivor-professional relationships. The range of dental interactions and the needs survivors described when receiving dental treatment are presented. Transcripts were analysed using NVivo software and thematic analysis methodology.

**Results**Three main themes were identified: the dental encounter ('it really panics me'); the opportunity to disclose; and choice and control.

**Conclusion**This is the first UK study to present qualitative data from CSA survivors about their experiences of dental care. Survivors wish to access dental care but tailored support is needed to ameliorate reminders of abuse and traumatic stress triggers. Trauma-informed care may address difficulties with treatment if dental staff adopt flexible approaches and work collaboratively with survivors to facilitate relational safety. (Please note, in this paper, 'survivors' refers to those sexually abused as children).

## Key points

- Survivors want to access dental care but encounter difficulties resulting from histories of child sex abuse.
- These difficulties can be overcome when dental practitioners and staff take account of the underlying psychological trauma.
- Trauma-informed dental treatment prioritises individualised care by offering choice and control.

## Introduction

The Crime Survey for England and Wales estimates 3.1 million people have been subject to child sexual abuse (CSA): 7.5% of adults aged 18-74.^1^ The figure for women is higher: one in six women reported unwanted sexual contact before the age of 16.^1^^,^^2^Therefore, a significant proportion of adult dental care patients will be survivors of CSA.

Dental practitioners offer oral care to all sectors of the population^3^ and act as gatekeepers to special care dentistry,^4^ which many survivors may need to access due to the link between abuse and poor oral hygiene in adults.Research suggests that many survivors experience significant difficulties at all stages of the dental interaction. After overcoming the difficulty of booking and attending an appointment, an underlying sense of helplessness is reported by survivor-patients through feeling disempowered and out of control in the dental chair.^5^^,^^6^ Specific aspects of the appointment, such as having implements in the mouth and feeling claustrophobic, link to past experiences of being in danger and pain.^7^ Overall, survivors may be observed by dental practitioners as anxious but their dental care experiences are often more complex in nature, as there is a link to previous trauma.

Trauma-informed approaches (TIAs) have been increasingly influential across healthcare settings,^8^^,^^9^ including dentistry,^10^ with calls for all healthcare professionals to receive appropriate training^11^ to address the iatrogenic harm caused by the lack of understanding of the effects of psychological trauma on healthcare treatment.^12^TIAs in dentistry in the UK are nascent but advances in treating psychologically traumatised patients in US dentistry positions TIAs as effective in meeting the needs of patients with psychological trauma.^10^ Traditional mental health approaches are organised around the question 'what is wrong with you?', whereas trauma-informed approaches are interested in 'what happened to you?'.^9^ The former question may close down conversations around the cause of distress, shutting out relevant information on trauma triggers. The TIA aims to open dialogue and gather information to structure care, which benefits dentists by facilitating the survivor to allow dental treatment.

There is evidence that using TIAs can enable the achievement of dental treatment aims^10^ without causing secondary, iatrogenic traumatisation^13^ and can contribute to recovery from primary trauma. The high proportion of CSA survivors in the general population has led to calls for mainstream dentistry to adopt TIAs.^14^ Qualitative exploration of experiences can contribute to an understanding of the effects of trauma on survivors' oral health and dental care experiences, which contributes to the foundations of a TIA.^10^ This paper aims to explore and analyse survivors' perspectives of dental practices to offer dental practitioners ways in which they might adapt treatment of survivor-patients for optimal outcomes.

## Methods

This paper draws from a broader study investigating survivor experiences of trust in accessing healthcare. Survivors of CSA are often reticent in disclosing abuse narratives,^2^ making gleaning accurate, in-depth research data challenging. The study adopted a survivor-research methodology,^15^ whereby the primary researcher is a survivor of CSA and such a shared history facilitated co-constructed explorations of trust issues.

A total of 17 survivors were recruited using a snowball method. The primary researcher introduced the study to two professionals working with survivors. They introduced the study to other professionals and survivors. Word-of-mouth introductions then generated a linking network out into peer-led survivor networks in the UK and Sweden; no incentive or reward was made. The sample comprised 13 women and 4 men, of which 15 were white (13 British, 2 Swedish) and 2 Black British.

Semi-structured interviews took place in locations agreed between the researcher and the survivor with the onus on the survivor feeling at ease and safe. The conversations were recorded, transcribed by the researcher and subjected to analysis using NVivo software. Thematic analysis,^16^ the widely accepted and rigorously scrutinised qualitative research methodology, was adopted to analyse the passages directly relating to dental encounters to identify themes.

The project received ethical approval from the University of Essex and addressed the safety and care of participants and the researcher. Local survivor stakeholders were consulted on the study focus and design. Participants' anonymity is assured through pseudonyms and disguising identifiable material. All participants consented to interview and to have their data included in published research. All data are anonymised.

## Results

### Theme 1: the dental encounter - 'it really panics me' (survivor of CSA)

Of the 17 participants, 12 reported avoiding dental care appointments:

- '*It's been really difficult […] I think, um, so for quite a long time I think I just avoided, so I didn't go to the dentist*' - Jo.

This was explained as a fear that oral contact would elicit somatic and interpersonal abuse memories, triggering trauma responses of extreme traumatic-agitation and combinations of fight, (for example, aggression), flight (for example, avoidance), freeze (for example, inability to respond), or dissociation (for example, fainting, or somatic derealisation). The keynote of psychological trauma is triggered memories being experienced as the abuse happening in the present moment. Survivors' stress-response behaviours, or verbal utterances, may appear to be out of proportion to the situation, especially to those who are not trauma-informed:

- '*I actually felt like I was going to pass out and it wasn't, it wasn't like an anxiety about feeling claustrophobic, I really felt it coming from somewhere deep inside, I felt, it was a memory*' - Helen.

One participant spoke of a traumatic triggering when an intrusive experience with a dentist reminded them of childhood abuse:

- '*It's like her hands or dentists hands, the ones that I have had in my face, they remind me of, they have the same kind of hardness of holding, it's not like gentle, it's like "crechhhh" more, it's like pushing and more like […] so it reminds me of my grandfather's hands which I have always remembered. I have, have always remembered the touch of his hands [grandfather: abuser], he was so hard with us, with touching us*' - Yasmin.

Sometimes the link between the abuse experience and resistance to dental care was implicit or metaphorically expressed:

- '*You're just little and it's very abusive with his big old fingers in your mouth*' - Tessa.

For others, the link between dental care and the original abuse was clear and known:

- '*Because of what happened as a child [CSA], I froze, so I just sat there frozen, and it meant that I stopped seeing that dentist as soon as I could*' - Rachel.

Survivors experienced a trauma triggering when procedures involving the mouth caused gagging or painful or sensitive sensations, which reminded them of the sexual abuse. This was mentioned recurrently by many participants:

- '*Anything that touched in my mouth hurt, so you know, I went for years without having an injection because that really hurt because it related to [CSA]*' - Stella.

Trauma resultant from CSA often results in combined life challenges, adding layers of difficulty to dental treatment:

- '*I haven't been able, since we were homeless. I did go, I mean it was hard but I went every six months for a long time but since homelessness…*' - Tessa.

Survivors who felt their psychological trauma was misunderstood felt stigmatised and shamed in their interactions with dental staff:

- '*They treated me like I was crazy […] the dentist, he, you know [...] ignorant is the word that I feel like*' - Milla.

Survivors described shame regarding poor oral health compounding the shame of CSA, especially when they are not given opportunities to explain:

- '*It's not that I don't look after my teeth, although I don't look after my teeth very well either, so it is partly that but it's also partly that I have really poor teeth because of her [mother] drinking AND then my brother damaged my teeth AND I didn't look after my teeth because of neglect but then you're treated like the onus is just all on you, like you've always been this fully responsible autonomous adult that should have been looking after your teeth*' - Caroline.

However, it is important to note that not all these survivors have issues with dental treatment:

- '*That is strange because that was a very tricky part to handle in therapy I would say, with the penis in the mouth, I think that's strange that I haven't had any problems with dentists*' - Frank.

### Theme 2: the opportunity to disclose

Many survivors avoid disclosure, for instance when asked if her dentist was aware of her history, Ruby responded *'no, god no!'*

Being able to disclose CSA is related to the life-course of the survivor and their location on the trajectory of recovery. When asked by the researcher, 'would you have welcomed being specifically asked by that previous dentist?', Jake replied:

- '*I don't think I would have been in a position when I was younger to actually tell people why, again I think a lot of it's come from my own knowledge and experience*'.

Some survivors could disclose fear even if they could not disclose the abuse*:*

- '*I always told them, I said I'm terrified*' - Tessa

However, disclosure is difficult in the absence of a facilitated opportunity to share fears or abuse histories and some participants described dental care as task focused:

- '*They just want to get on with their job and if anything, you're just an inconvenience*' - Milla.

In these instances, it was difficult to notify the dentist of fears or trauma histories:

- '*I went to a dentist who was supposed to be very patient-sensitive to working with people who are nervous so I said "look, I've got a dental phobia" and he said "yeah, ok, yeah that's fine, we deal with loads of patients who are quite, all the time, stuff keeps coming back" and that was it, that was the conversation*' - Jake.

The dental questionnaire was one gateway towards disclosure as it allowed an iterative discussion:

- '*When you go in the dentist and they ask you for diagnoses I was then able to put PTSD down so she asked me how did you get that and I was "oh sexually abused" and she was really understanding*' - Milla.

To facilitate partial disclosure, one survivor had a suggestion:

- '*As we know that people find it difficult to verbalise, if people had a card that just said, "please note if I'm having [other examples] a dental treatment that I have a history of trauma"*' - Jake.

Enabling discussion, to the extent to which the survivor wishes, allows the sharing of triggers and fears creating dental care that feels safe:

- '*He [dentist] was very very good. I didn't have to go into it all, I've just said I've had experiences when I was younger, I don't like people over my face, that's all I had to say to him*' - Chloe.

Sometimes the opportunity to disclose, and the willingness, takes time:

- '*I've actually had her for five years and she's only just not long found out and I've felt like I've built up this trust with her and she really understands*' - Milla.

Dependent upon the life-course and relational safety, some survivors do want to, and are able to, disclose their histories:

- '*The last dentist I went to, I said "look I've got a dental phobia" […] bearing in mind I'm mid-fifties, she is the only dentist in my whole life that's ever asked me why I have a dental phobia. I said I was sexually abused, so, she said "well, thank you for telling me that, that can't be easy for you*"' - Jake.

### Theme 3: survivor choice and control

Survivors wanted dentists to allow them choice over treatment options and support and some said they would insist on getting what they needed, while others were less able to ask, dependent on the stage of recovery from trauma.

This participant wanted to be asked:

- '*What can I do to make you feel more comfortable, or make you feel trust in me*' - Yasmin.

Individualised choice enabled treatment:

- '*He'd let me lie on my side*' - Chloe
- '*There's been times, like I'm an adult, but at the dentist I still have a nurse hold my hand*' - Milla.

Without these options survivors could be triggered:

- '*It's like, with the dentist, it's like this intrusion again, no control, totally like left out, ergh, just being [abused]*' - Yasmin.

Some participants had self-help techniques, such as rhythmic breathing. Others used a form of helpful dissociation to cope and needed the dentist to understand when they needed to withdraw:

- '*I had to go into a kind of internal meditation because once they put that big dam in like that it spreads your mouth open wide […] that just made me kind of feel like I needed to go inward and just be quiet and they were kind of chatting […] he'd sort of sometimes tell me to, you know, close your mouth a bit or open it up a bit and he just felt so far away, that I thought he was still talking to his assistant*' - Ruby.
- '*I think for me, it definitely becomes probably a dissociative thing, it's the only way I can get through it*' - Jo.

Survivors felt safer when they had the means to stop the treatment if they were becoming triggered:

- '*I'm always in full control with her and that's the thing, always in full control, and she'll say put your hand up and stop*' - Helen.

This included the pace of relationship building before invasive procedures:

- '*Basically she built the rapport up […] the first session we had a little chat; second, check session, just sat in the chair, think I had a little check-up; third session, fillings started*' - Helen.

Continuity of staff facilitated a trust which ensured the continued pace of treatment:

- '*And now she's gone and that makes me nervous about going back because I feel like I'm starting all over again and you might not necessarily get people who you think are sympathetic' -* Milla.

Choice and control extended to the physical and relational proximity of dental staff; CSA involves a person in a position of responsibility breaking boundaries, therefore any repeat of unexpected, unwanted or inappropriate proximity can be triggering:

- '*I've had experiences when I was younger, I don't like people over my face*' - Chloe
- '*One dentist used to put the mat over my chest and be grappling […] and I thought his touching was inappropriate, but because of what happened as a child*' - Rachel.

Relational proximity refers to survivors' need for dentists to be approachable, warm, kind and friendly:

- *'She could be so much more considerate and, and soft in her touch*' - Yasmin.

There was a desire for a trusting relationship where vulnerabilities on both sides can be aired, as this would communicate a genuine desire to help:

- '*Because it would also show me her [dentist] vulnerability, so that would make me feel safe that she would come to me and say, "you know I just learnt something, I got this email from wherever and reminded us, all dentists, about this situation, would you, could you forgive me for not asking you", that would make me feel really really good actually, that would make me more relaxed*' - Yasmin.

It is clear from these data that when disclosure was facilitated and staff responded sensitively by offering choice and control, the dental encounter held less fear of re-traumatisation of triggered stress responses, which enabled better treatment outcomes.

## Discussion

Dental care, while understood to be necessary, represented a major challenge for many participants because of its interpersonal and sensory parallels with CSA. Based on these findings, dental treatment presents a risk of triggering a psychological trauma response impacting access to oral healthcare for many survivors of CSA.^17^^,^^18^

The themes of panic in the dental encounter, dilemmas around disclosure and choice and control, indicate that a multitude of difficulties are present for survivors attempting to access the dentist. This supports the findings from other studies^5^^,^^6^^,^^7^ and contributes to the conclusions of other studies that there is a link between CSA and dental fear.^5^^,^^7^^,^^19^^,^^20^

The current study also links with broader dentistry research which considers the specific needs of patients with complex mental health needs, including those with traumatic stress. A recent study^21^ found patients with long-term mental illness required specific practical and emotional support to access dental care, a finding in keeping with the results of this study that emphasise the need for patient-led adaptation to treatment, additional time for discussion and safe ways of stopping treatment if the patient is triggered. TIAs can be utilised to provide a framework for these additional support needs that could be of benefit to patients with complex mental health needs, irrespective of whether they have a trauma history.^13^ The evidence of the effectiveness for TIAs is mixed but there is some evidence for its applicability to dental care.^8^^,^^10^ One aspect of TIAs that would be crucial based on the evidence presented here is the use of education for practitioners to better understand psychological trauma.^16^

Disclosing CSA is difficult,^2^ yet survivors in this study expressed the need to have their psychological trauma understood, even if full disclosure was not possible. Facilitation of disclosure includes careful wording of pre-treatment questionnaires, sensitive responses to survivor remarks, or the use of a trauma-card, much like an organ-donor card. Disclosure of child abuse history is an issue in other healthcare settings^2^ and this study suggests that oral health practitioners might need additional training to understand the specific issues with disclosure of CSA. However, as this study showed, full disclosure was not always necessary or desirable; a general awareness of psychological trauma and its impact on dental care was a good starting point for many survivors.^10^^,^^12^^,^^22^

This study also found that intersectionality of CSA combined with other forms of social justice inequalities create strata of psychological trauma. These inter-woven layers create an aggregated set of challenges in accessing dental care.^23^ As indicated by some of the participants' accounts of homelessness, childhood neglect, physical abuse to the mouth and oral self-neglect, there may be multiple, interacting difficulties that contribute to survivors' experiences with oral care. An intersectional approach is required to consider the interacting experiences that contribute to the prevalence of poor oral health and the psychological difficulties associated when using dental services. In spite of these challenges, this study found evidence that CSA survivors and dental professionals can create therapeutic working relationships that can enable safe, non-retraumatising access to dental care. Dental practitioners in the UK need to be supported to engage with people who have experiences of CSA that impact their ability to access dental care. Likewise, CSA survivors themselves need to be made aware that dental practice can be trauma-informed to encourage engagement and that the range of experiences a person will have when visiting a dental practice will vary and that not all forms of oral health treatment are invasive.

This study benefited from the trust between researcher and participants in their shared histories of CSA, which many participants spoke of during interviews. With regard to limitations, comments about dentistry were woven through the transcripts, as the study did not set out to explore dental experiences *per se,* but only in regard to survivor narratives of trust and trustworthiness. Further research building on these initial findings might seek to verify and expand on these initial findings. Furthermore, the sample was gathered through a snowballing method to find survivors who wanted to talk about trust; a sample wishing to explicitly speak of oral health and treatment experiences might draw a different profile of participants. Studies focusing on intersectionality of CSA survivors or special care dentistry are also areas for future research.

## Conclusion

This is the first UK study to present qualitative evidence from CSA survivors about their experiences of accessing dental services.

Trauma responses triggered by dental treatment may appear out of proportion to the situation but awareness of the distinct challenges CSA survivors face in receiving dental care and the facilitation of disclosure, choice and control can mitigate these barriers.

Trauma-informed dental care understands that CSA can cause strong emotional and physiological responses in adulthood. Dentists able to provide trauma-informed treatment options create opportunities for both dentists and patients to gain from this approach through more easeful appointments and better outcomes for oral health.
